# Urban geochemical changes and pollution with potentially harmful elements in seven Russian cities

**DOI:** 10.1038/s41598-020-58434-4

**Published:** 2020-02-03

**Authors:** Andrian A. Seleznev, Ilia V. Yarmoshenko, Georgy P. Malinovsky

**Affiliations:** 1 Institute of Industrial Ecology, Ural Branch of Russian Academy of Sciences; Postal address: S. Kovalevskoy, Str., 20, 620219 Ekaterinburg, Russia; 2 Ural Federal University named after the first President of Russia B. N. Yeltsin, Postal address: 19 Mira Str., 620002 Ekaterinburg, Russia

**Keywords:** Ecosystem services, Urban ecology, Environmental impact, Environmental impact, Sustainability

## Abstract

This paper presents results of an analysis of potentially harmful elements (PHEs, Pb, Zn and Cu) and conservative element (CE, Fe) concentrations in urban surface deposited sediment (USDS). The study was conducted in seven large Russian cities located in different geographic and climatic zones, and in territories with different geology and anthropogenic pressures: Chelyabinsk, Magnitogorsk, Nizhniy Novgorod, Nizhniy Tagil, Rostov-on-Don, Tyumen, and Ufa. The initial geochemical baseline relationships between PHEs and CE concentrations in the USDS were reconstructed for each city applying an approach based on linear weighted fitting of PHE as a function of CE with lower weights assigned to more polluted samples. The reconstructed average initial baseline Pb, Cu, and Zn concentrations varied between 17–52, 25–196, and 91–413 mg kg^−1^, respectively. Several new criteria for assessing the degree of geochemical transformation and pollution of the urban environment, such as the percentage of polluted samples, average pollutant concentration in polluted samples, and weighting degree index δ, were suggested and compared with common criteria, such as the PHE concentration and the geo-accumulation index. The environmental rank of a city significantly differed depending on whether the criterion for ranking was total PHE pollution or changes in comparison with the initial geochemical baseline.

## Introduction

An urban landscape is an environment formed as an upper geological strata of the Anthropocene^1,2^. The content of elements in environmental compartments in an urban area changes as a result of anthropogenic activities during landscape forming. The transformation of geochemical conditions in an urban area is a permanent environmental process governed by natural and artificial components. The content of elements in compartments of the urban environment at some initial moment, that either existed in reality or is assumed, before artificial and natural components of the environment were subject to any anthropogenic impact after creation of the landscape, is considered as initial geochemical baseline (IGB) conditions.

Geochemical transformation and change of the IGB is a complex process affecting all components of the urban environment^3^. The primary driving forces of the geochemical transformation include pollution of the environmental compartments by point and nonpoint sources^4^ and erosion and degradation of the environmental compartments as a result of natural and anthropogenic impacts^5^.

Geochemical transformation in the urban environment consists of altering mineral and elemental composition, modification of physicochemical properties of the urban soil^6^, urban surface deposited sediment (USDS) formation, in particular road deposited sediment^4,5,7^, changes in volume of the surface stormwater runoff^8,9^, redistribution of pollutants^3,10^, and forming geochemical barriers^7^ among others.

USDS is an environmental compartment that is involved with the majority of geochemical transformation processes. In the urban environment, the rate of sediment yield increases significantly in comparison with forest and agricultural land uses. Urban surface deposited sediment is formed as result of soil erosion processes, abrasion of various structures, and deposition of sedimentary material over various surfaces^5,11^. A model of an urban sedimentary cascade should describe transport and accumulation of particles and pollutants at all stages of the sedimentation processes from the sources of the sediment to their ultimate deposition^7^.

While sediments are associated with surface layers of soils, USDS is more polluted by the atmospheric precipitations and wastes than other environmental compartments in urban area^7,12,13^. Fine particles may accumulate pollutants, pathogens, viruses, and other potentially harmful elements (PHEs)^14^. Thus, the sediments represent a secondary and nonpoint pollutant source and represent an important environmental risk due to the high contribution of the total dust fraction and pollutants in urban environments.

Surface deposited sediment is often used as an indicator in urban geochemical studies^15–19^. Sediments are lithologically and geochemically associated with the territory of the city, thus they reflect the geochemical conditions of the surrounding area. The sediment signs the pollution and its pathways in urban environment^20,21^. Assessing the IGB and subsequent changes of geochemical conditions in cities are important factors when conducting environmental geochemical studies in urban areas^19,22^.

Earlier, an approach was proposed to reconstruct initial geochemical conditions in an urban area^19^ which allow to assess the geochemical transformation of the urban environment quantitatively. The method was based on analyzing the relationship between PHE and conservative elements (CE) in the USDS. The approach uses a linear weighted fitting of the PHE-CE relationship with lower weights assigned to more polluted samples^19^. Strong mineralogical reasons of the linear regression between concentrations of two elements in soils and sediment samples were suggested by Van der Veer^23^.

In this study, an analysis of geochemical conditions for PHEs (Pb, Zn, and Cu) and a CE (Fe) in the USDS was conducted for seven large cities in the Russian Federation. The cities were located in different geographic and climatic zones and included territories with different geology and anthropogenic pressures. The aims of the current study were to (i) reconstruct the initial baseline relationship between PHEs and the CE concentrations in USDS for each city with the approach suggested by Seleznev *et al*.^19^, (ii) assess the degree of pollution and the degree of geochemical changes in the cities, and (iii) rank the cities by the degree of pollution and the degree of geochemical transformation.

## Materials and Methods

### Sample collection and preparation

The object of the study is contemporary geochemical conditions represented by PHE and Fe concentrations in the USDS. Samples of USDS were collected at natural surface geochemical traps such as surface depressed zones of the microrelief inside blocks of houses, in particular from puddles. The USDS formation mechanism was previously described in detail^24,25^. Locally depressed surface areas in residential districts are filled with sedimentary material washed out and transported from surrounding landscape zones. This sediment contains atmospheric dust, solid and suspended particles of soil, erosion and other materials. The catchment area consists of building roofs, grounds, pavements, local roads within the district, green zones, and other water harvesting surfaces. The solid material of the sediments is naturally intermixed. Pollutants absorbed on the particles are transferred and accumulated in the USDS as well. The typical thickness of the sediments is about 5 cm. The fine sedimentary material is constantly transported over the area of the block and redistributed by surface rainwater runoff and vehicle wheels. USDS accumulate pollutants over space and time within the area of the residential yard and provide an integrated reflection its current geochemical conditions.

**Figure 1:**
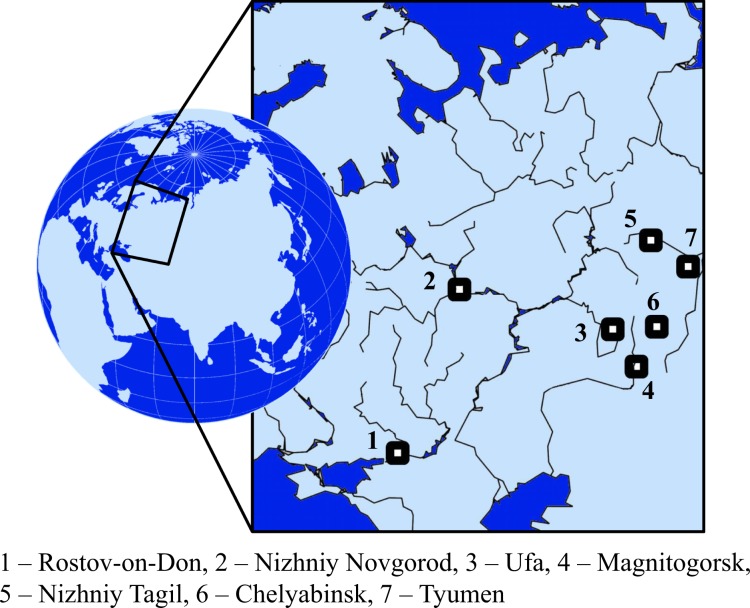
Location of surveyed cities in Western part of Russia.

USDS surveys were conducted in seven Russian cities located in different geological, geographic, climatic, and industrial zones (Figure 1). Intensive urbanization with rapid population growth and the development of new residential districts has occurred in the studied cities over less than the last 100 years. Short descriptions of the chosen cities are presented in Table 1.

**Table 1:** Descriptions of the surveyed cities.

City population, million people/cars per 10^3^ people	Geographic and climate zone, average temperature, °С, Jan/Jul	Geological features^35^	Industries
Tyumen 0.77/363	The Western Siberia, forest taiga zone with waterlogged areas, temperate continental climate -15/18.8	West-Siberian plain, Tyumen downwarp; diorites and gabbros of formations of Prejurasic age; loams, clays, silts and lake-alluvium of the Upper Pliocene and Holocene age	Metal processing, machinery, oil processing, gas-fired power plants
Chelyabinsk 1.2/269	The South Urals, forest-steppe zone, temperate climate -14.1/19.3	East Urals uplift and West Side of West-Siberian plate; granites, diorites, coals, limestones, sandstones, dolomitic limestones of formations of Prejurasic age; sands, siltstones, loams, alluvial sediments of floodplain terraces, pebbles, gravels, and eluvial-deluvial sediments of the Upper Pliocene and Holocene age	Ferrous and non-ferrous metallurgy, chemical industry, machinery, coal-fired power plants
Nizhniy Tagil 0.36/240	The Middle Urals, mountain-forest zone, temperate continental climate -14.5/17.8	Middle Urals, Tagil megazone; harzburgites, serpentinites, basalts, green schists, mica-quartz and graphite-quartz schists, diorites, gabbros, andesites, dacites of formations of Prejurasic age; eluvial and deluvial sediments, clays, sandy loams, alluvial sediments of floodplain terraces, pebbles, sands, and loams of the Upper Pliocene and Holocene age	Ferrous metallurgy, coking, machinery, chemical industry, production of building materials
Magnitogorsk 0.42/297	The South Urals, steppe zone, sharply continental climate -14.1/19.2	South Urals, West Magnitogorsk zone; trachibasalts, trachiriolites, basalts, andesites, rhyodacites, lavas, and clastolavas of formations of Prejurasic age; alluvial sediments of floodplains, clays, sands, peat, deluvial sediments, eluvial-deluvial sediments, and limes of the Upper Pliocene and Holocene age	Ferrous metallurgy, metal processing, gas-fired power plant
Ufa 1.1/278	Forest-steppe zone, temperate continental climate -12.4/19.7	Volga-Ural Anteclise, Verkhnekamsk basin; gypsum, anhydrite, sandstone, marl, siltstone, dolomite, limestones of formations of Prejurasic age; alluvium, koluvium, deluvium, sandstones, sandy loams, loams, clay of the Upper Pliocene and Holocene age	Oil processing, oil chemical industry, machinery
Rostov-on-Don 1.1/285	Steppe zone, moderate continental climate -3/23.4	East European plate, Rostov ledge; sands, clays, gravel, pebbles of the Lower Pliocene Limestones, shells, siltstones, marls of the Upper Miocene; alluvium floodplain terraces, sands, pebbles, loams, sandy loam, eluvial and proluvial sediments of the Upper Pliocene and Holocene age	Machinery, river shipping, food industry
Nizhniy Novgorod 1.3/276	Broad-leaved forests, mixed forests and taiga zone. Humid continental climate -8.9/19.4	Volga-Ural Anteclise, clays with interbeds of siltstone, sand with gravel of sedimentary rocks, siltstone, loam, marl, gypsum, limestones, dolomites of prequarternary age; alluvial sediments, sands with gravel, loam, clay, eluvial and solifluction formations, sands, eluvial and deluvial formations of the Holocene age	Machinery, river shipping

Samples of USDS were collected in residential areas of the cities during the summer seasons in 2016 (Tyumen, Chelyabinsk, and Nizhniy Tagil), 2017 (Ufa and Magnitogorsk), and 2018 (Rostov-on-Don and Nizhniy Novgorod). The majority of the population in surveyed cities live in districts of multi-story apartment buildings and the sampling sites were located in the courtyard areas that were surrounded by a block of such houses. The area of surveyed blocks varied in the range of 20,000–60,000 m^2^. Besides residential dwellings, the residential districts comprise the local public infrastructure that includes hospitals, schools, kindergartens, malls, recreational areas, and parks. These residential blocks in the cities surveyed had the same construction features and the same set of landscape sites within the block, including green zones, passages, and children’s playgrounds.

The sampling sites were chosen on an irregular grid within the territory of each city. The method of Seleznev *et al*.^25^ and Seleznev and Yarmoshenko^24^ was used to collect USDS samples. Roads with heavy traffic and industrial plants were located at a considerable distance from the studied sites (500–3000 m), and roads with medium traffic intensity were located at a distance of approx. 100 m from the sampling sites. A member of the research team visited the residential yard and selected depression sites where surface deposited sediment had formed. Samples were taken using a sampling scoop from the upper 5 cm layer. Usually, 3–5 samples were collected from a depression sites in a courtyard area and the samples were then combined into one composite sediment sample. The total sample mass was approx. 1.5 kg (dry weight). Domestic wastes and debris were removed during the sampling process. Information on the location and site-specific descriptions of sampling sites were documented. Sampling sites and samples were photo documented.

The USDS samples were air-dried at ambient temperature in the laboratory. The dried samples were crushed, homogenized, and thoroughly mixed. The coarse particles ( > 3 mm) of debris, tree branches, roots, stones, and gravel were discarded. Then a representative subsample of 20 g was collected for analysis. The subsample was abraded to powder in an agate pounder.

### Chemical analysis

The chemical analysis of the samples was performed at the Chemical Analytical Centre of the Institute of Industrial Ecology. The total PHEs concentrations in the samples were determined with inductively coupled plasma mass spectrometry (ELAN 9000; Perkin Elmer Inc., USA). Sample preparation and analysis procedures were conducted according to the technique for measuring metal content in solid objects by spectrometry with inductively coupled plasma certified by The State Bureau for Environmental Protection of the Russian Federation^26^. Solid samples were prepared to determine the total element contents using extraction with three acids (HNO3, HClO4, and HF). The sample preparation method was similar to the United States Environmental Protection Agency (US EPA) method EPA-821-R-01-010^27^. Measurement quality control was ensured by using certified methodologies and by accreditation of the Chemical Analytical Centre of the Institute of Industrial Ecology by The Russian System of the State Accreditation Laboratories.

### Reconstruction of initial geochemical baseline conditions

The IGB relationship between PHE and CE concentrations was reconstructed using the method described in Seleznev *et al*.^19^. According to this method, the IGB relationship was defined by the linear equation:

1 $$Me0(Fe)=A\cdot Fe+B,$$

where Me0(Fe) is the PHE baseline concentration in mg kg^−1^ as a function of Fe concentration in g kg^−1^, and A and B are the regression coefficients.

The regression coefficients were fitted based on the results of measurements of PHE and CE concentrations in USDS samples, taking into account weights assigned in accordance with the equation:

2 $${W}_{i}=1/{({y}_{i}-0.95\cdot {\rm{\min }}{(y)}_{i})}^{\delta },$$

where δ is the dimensionless degree index, *y*_i_ is observed PHE concentration in i-th sample, min(*y*)_i_ is the minimum PHE concentration among the ten values closest to *y*_i_ in the sample dataset ranked by Fe concentration (see Seleznev *et al*.^19^ for more details).

In Seleznev *et al*.^19^ the δ-index was associated with the degree of pollution in city sediments. In the case of an absence of pollution δ = 0 and its value increases with greater PHE emission to the environment. Consistency between the results of the reconstruction of the IGB relationship with PHE and CE concentrations by this method and using the Cs-137 chronological approach was achieved when δ values were in the range of 0.5–0.75 in Ekaterinburg^19^.

This paper proposes a more formalized approach to weighting and selection of the δ-index. Iterative calculations of the linear dependence (1) were performed for δ = 0, 0.1, 0.2 … with increment of 0.1. Deviations, ΔMe_i_, of the observed PHE concentration, Me_i_, from the baseline concentration, Me0(Fe_i_), at the observed iron concentration, Fe_i_, for each sample are calculated. For each examined δ, samples are ranked according to obtained ΔMe_i_ (subindex j is used for ranked samples). Then, the number of samples, P, is determined, for which the following condition is true:

3 $$abs(\mathop{\sum }\limits_{j=0}^{P}\Delta M{e}_{j})=min.$$

The samples satisfying condition (3) are considered as unpolluted. Then the numbers of samples with ΔMe_j_ < 0 and ΔMe_j_ > 0 are estimated. The desired value of δ-index is chosen so that the total number and average deviations of the observed PHE concentrations from those predicted by the model (1) were about the same for unpolluted samples lying below and above the line corresponding to the IGB relationship.

In each city, the average pollution and average pollution of polluted samples were estimated as the ratio of the sum ΣΔMe_i_ to the total number of samples and the number of polluted samples, respectively.

Substituting the average observed Fe concentration into Eq. (1), the average concentration of PHE under the IGB relationship was estimated, Me0. The PHE concentration calculated in this way corresponds to the conventional background concentration. This concentration is also applicable for the calculating the geo-accumulation index (Igeo) using the equation^28^:

4 $${\rm{Igeo}}=lo{g}_{2}(\frac{{\sum }_{i}M{e}_{i}/N}{1.5\cdot Me0}).$$

## Results

In total, 335 samples of USDS were collected from seven cities in the period 2016–2018. The total number of samples in each city varied from 40 to 69.

According to site-specific descriptions, the landscape at the all surveyed residential areas was artificially created as a result of construction activities. The sedimentary material at the studied sites was formed from the soil erosion process and surface destruction of pavements, asphalt, and building construction materials (i.e., surfaces of roofs and walls). The base surfaces represented a variety of materials such as urban sealed soil and ground, anthropogenic fill, turf, asphalt, and different pavement surfaces. In the studied cities the surface sediments had a loose homogeneous structure, and they were visually identified easily and distinguished from the base surface substrate.

Based on the visual inspection of the sampling sites, the majority of the samples collected in Nizhniy Tagil, Chelyabinsk, and Magnitogorsk contained particles of metallurgical slag. The granulated slag was used instead of gravel as backfill to create drainage in landscaping and urban planning. During the survey, ground excavation works as well as landscape planning activities (e.g., greening, soil replacement) were observed in some sites. Cars were parked in all of the studied courtyards and there were both organized and unorganized (when the car is parked on the lawn) parking lots and intra-yard passages. Approximately 20% of the vehicles were illegally parked in unorganized parking lots of the yards. By visual inspection, intra-yard roads and parking lots were estimated to account for up to one third of the courtyard area.

Table 2 presents the results of chemical analysis of the sediment samples. The table contains the number of samples in each city and the average concentration of Fe, Pb, Cu, and Zn.

**Table 2:** Observed concentrations of metals (arithmetic mean for Fe, geometric mean for other metals).

City	Year of sampling	The number of samples	Fe, g/kg	Pb, mg/kg	Cu, mg/kg	Zn, mg/kg
Chelyabinsk	2016	60	28	66	50	338
Magnitogorsk	2017	41	32	37	51	289
Nizhniy Novgorod	2018	40	13	21	33	147
Nizhniy Tagil	2016	69	68	73	264	468
Rostov-on-Don	2018	40	16	32	31	169
Tyumen	2016	42	16	46	30	122
Ufa	2017	43	21	42	56	158

**Figure 2:**
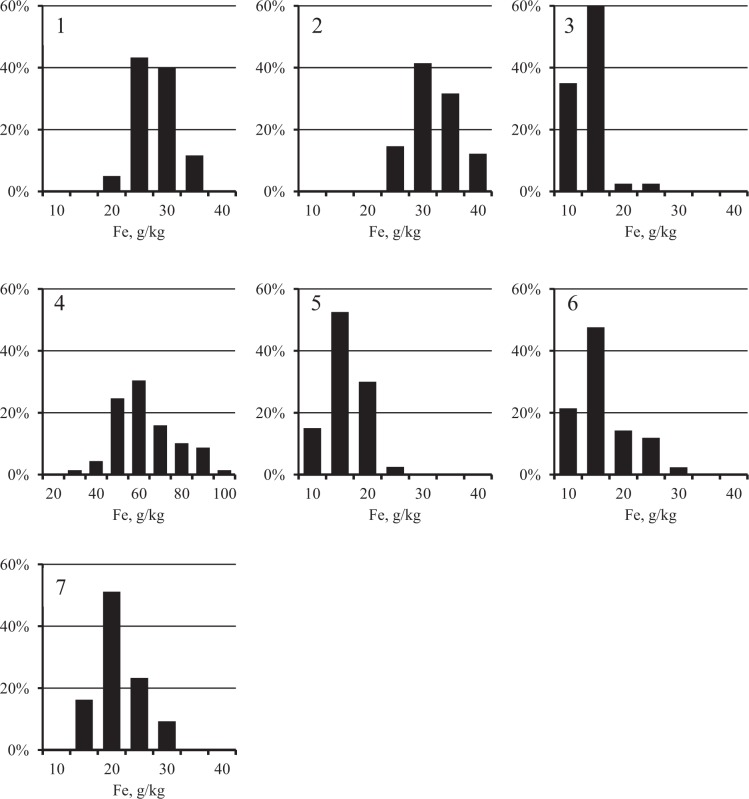
Distribution of Fe concentration in the samples of USDS in cities: 1 – Chelyabinsk, 2 – Magnitogorsk, 3 – Nizhniy Novgorod, 4 – Nizhniy Tagil, 5 – Rostov-on-Don, 6 – Tyumen, 7 – Ufa.

**Figure 3:**
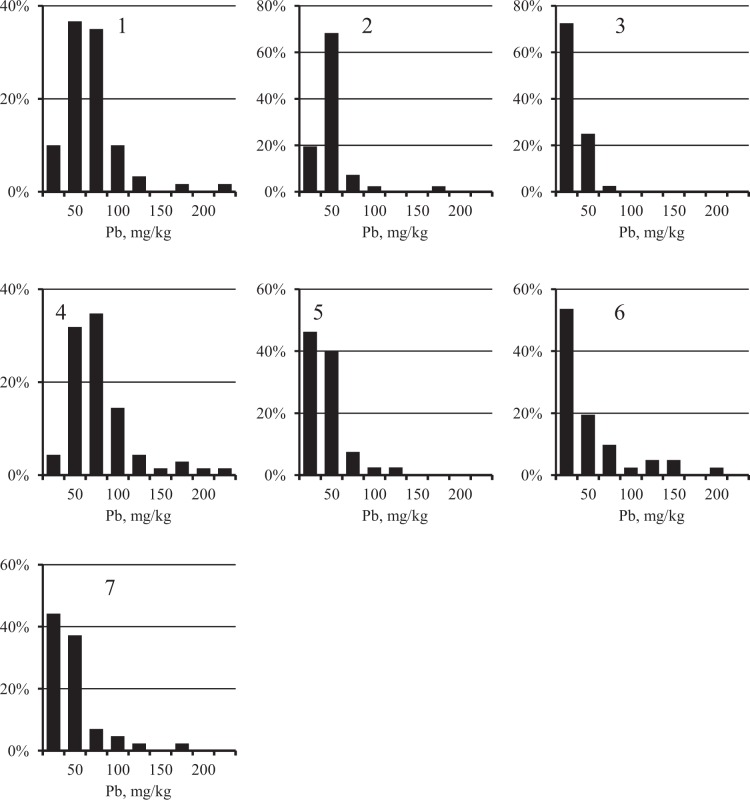
Distribution of Pb concentration in the samples of USDS in cities: 1 – Chelyabinsk, 2 – Magnitogorsk, 3 – Nizhniy Novgorod, 4 – Nizhniy Tagil, 5 – Rostov-on-Don, 6 – Tyumen, 7 – Ufa.

**Figure 4:**
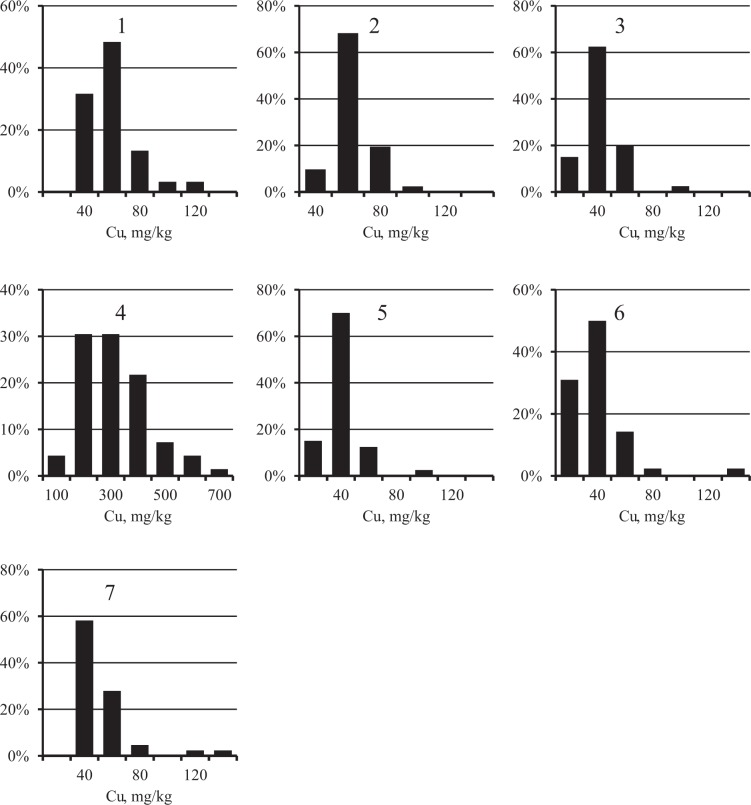
Distribution of Cu concentration in the samples of USDS in cities: 1 – Chelyabinsk, 2 – Magnitogorsk, 3 – Nizhniy Novgorod, 4 – Nizhniy Tagil, 5 – Rostov-on-Don, 6 – Tyumen, 7 – Ufa.

**Figure 5:**
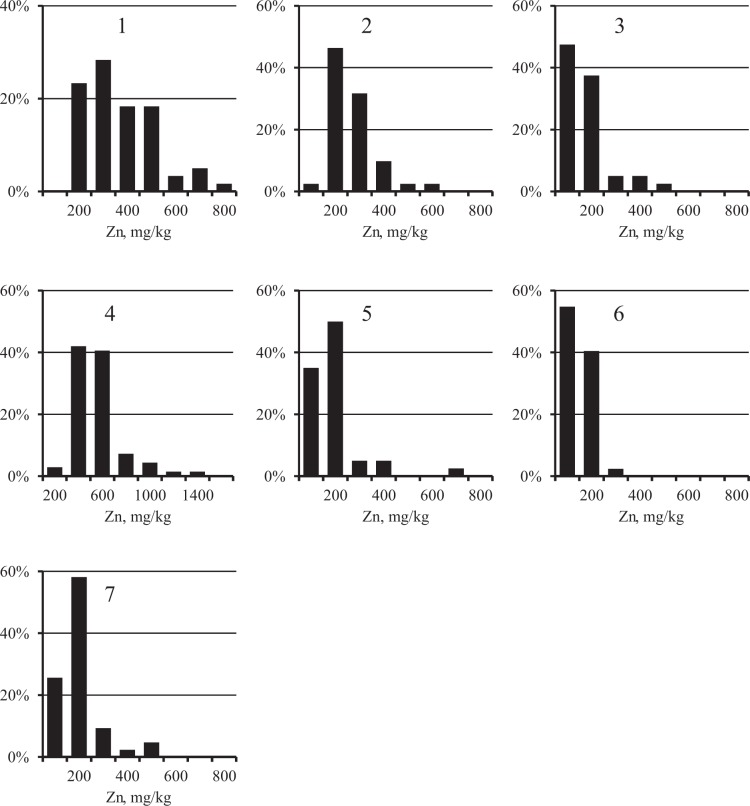
Distribution of Zn concentration in the samples of USDS in cities: 1 – Chelyabinsk, 2 – Magnitogorsk, 3 – Nizhniy Novgorod, 4 – Nizhniy Tagil, 5 – Rostov-on-Don, 6 – Tyumen, 7 – Ufa.

The distribution of Fe concentrations in each city was close to normal (Figure 2, Chi-square criterion, *p* > 0.05). At the same time, as expected, the distribution of PHE (Figures 3–5) was substantially asymmetric with a shift toward high concentrations. The cities surveyed differed in the average Fe content in the surface sediment. The highest Fe concentrations were found in Nizhniy Tagil, Magnitogorsk, and Chelyabinsk, while the lowest were in Rostov-on-Don, Tyumen, and Nizhniy Novgorod. For all PHEs, maximum concentrations were observed in Nizhniy Tagil.

**Figure 6:**
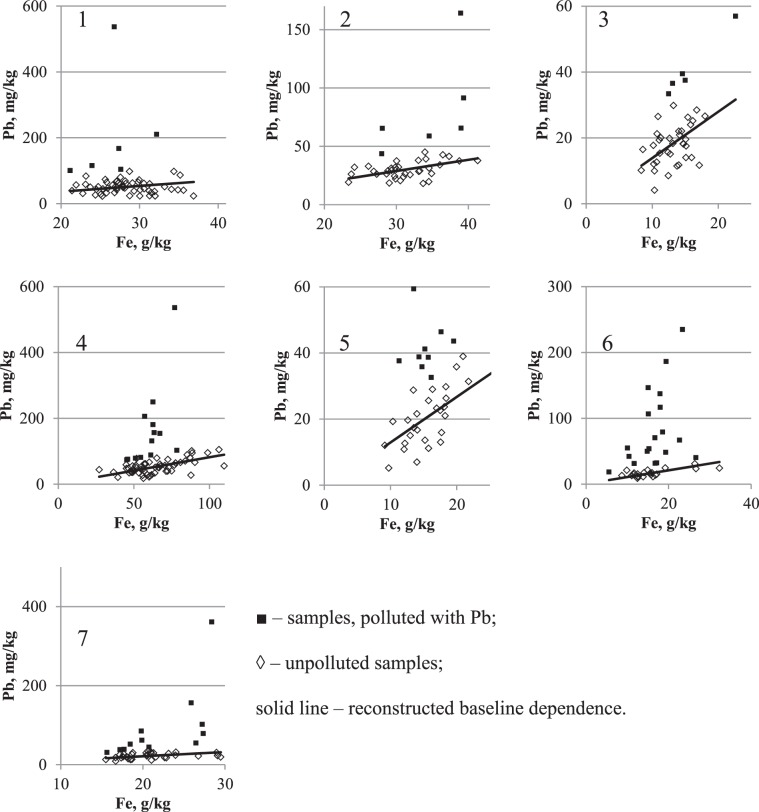
Reconstruction of baseline relationship between Pb on Fe concentrations in urban surface sediment in seven cities: 1 – Chelyabinsk, 2 – Magnitogorsk, 3 – Nizhniy Novgorod, 4 – Nizhniy Tagil, 5 – Rostov-on-Don, 6 – Tyumen, 7 – Ufa. Vertical axis – Pb concentration, mg/kg; horizontal axis – Fe concentration, g/kg.

**Figure 7:**
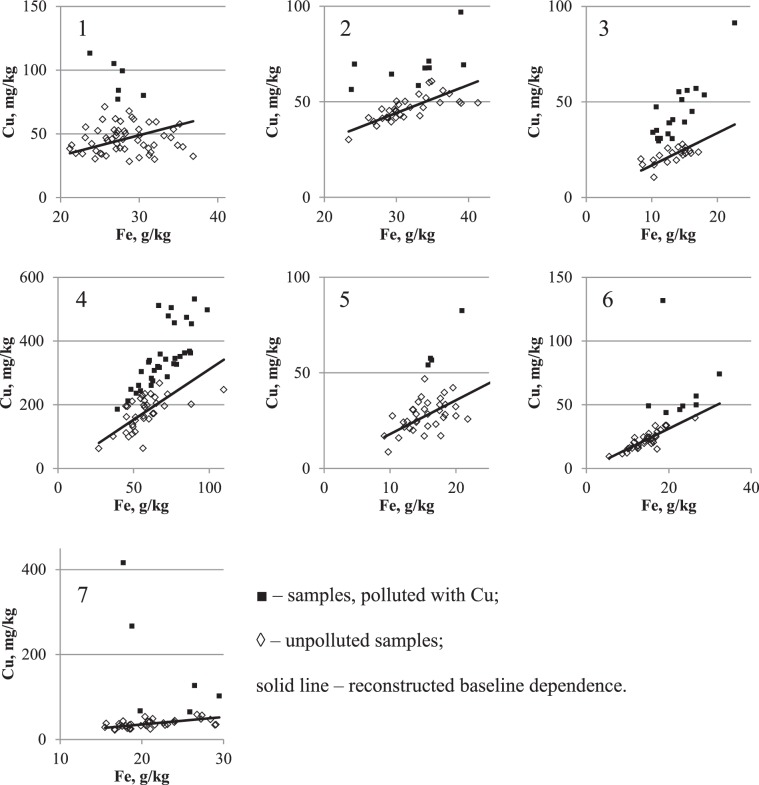
Reconstruction of baseline relationship between Cu on Fe concentrations in urban surface sediment in seven cities: 1 – Chelyabinsk, 2 – Magnitogorsk, 3 – Nizhniy Novgorod, 4 – Nizhniy Tagil, 5 – Rostov-on-Don, 6 – Tyumen, 7 – Ufa. Vertical axis – Cu concentration, mg/kg; horizontal axis – Fe concentration, g/kg.

**Figure 8:**
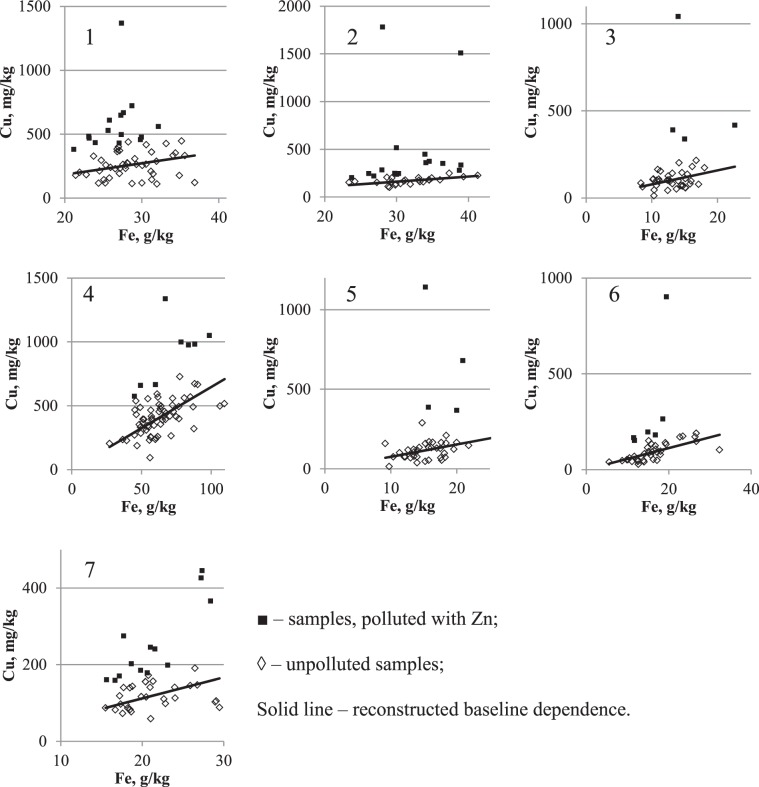
Reconstruction of baseline relationship Zn on Fe concentrations in urban surface sediment in seven cities: 1 – Chelyabinsk, 2 – Magnitogorsk, 3 – Nizhniy Novgorod, 4 – Nizhniy Tagil, 5 – Rostov-on-Don, 6 – Tyumen, 7 – Ufa. Vertical axis – Zn concentration, mg/kg; horizontal axis – Fe concentration, g/kg.

Analysis results of the relationship between concentrations of PHE and Fe in the cities are presented in Figures 6–8 the form of scatterplots. These figures show samples for which the presence of pollution or its absence was established. The line in the figures represents the reconstructed IGB level with the δ coefficients listed in Tables 3–5.

**Table 3:** Calculated coefficients of weighted linear dependence between the concentrations of Fe and Pb.

City	δ	A	SE	B, mg/kg	Mean Pb baseline concentration, mg/kg
Chelyabinsk	0.36	1.75	0.10	1.3*	50
Magnitogorsk	0.53	0.95	0.03	−0.2*	31
Nizhniy Novgorod	0.25	1.34	0.07	−0.2*	19
Nizhniy Tagil	0.49	0.81	0.03	0.8*	52
Rostov-on-Don	0.6	1.33	0.08	−0.9*	21
Tyumen	1.1	1.01	0.06	0.6*	17
Ufa	0.65	1.05	0.06	−0.2*	23

*Insignificant, p > 0.05.

**Table 4:** Calculated coefficients of weighted linear dependence between the concentrations of Fe and Cu.

City	δ	A	SE	B, mg/kg	Mean Cu baseline concentration, mg/kg
Chelyabinsk	0.37	1.59	0.06	0.9*	46
Magnitogorsk	0.87	1.46	0.03	0.2*	47
Nizhniy Novgorod	1.05	1.66	0.06	−0.1*	23
Nizhniy Tagil	0.9	3.02	0.12	−5.3*	196
Rostov-on-Don	0.35	1.75	0.08	0.2*	28
Tyumen	0.7	1.54	0.04	−0.6*	25
Ufa	0.6	1.73	0.06	0.3*	37

*Insignificant, p > 0.05.

**Table 5:** Calculated coefficients of weighted linear dependence between the concentrations of Fe and Zn.

City	δ	A	SE	B, mg/kg	Mean Zn baseline concentration, mg/kg
Chelyabinsk	0.47	8.91	0.50	5.2*	256
Magnitogorsk	1.05	5.32	0.18	0.1*	170
Nizhniy Novgorod	0.35	7.76	0.55	−2.3*	106
Nizhniy Tagil	0.36	6.35	0.22	1.7*	413
Rostov-on-Don	0.35	7.43	0.54	−2.9*	120
Tyumen	0.46	5.67	0.30	0.2*	91
Ufa	0.64	5.42	0.29	2.3*	119

*Insignificant, p > 0.05.

Tables 3–5 present the calculated coefficients of weighted linear dependencies between the concentrations of Fe and Pb, Cu, and Zn, respectively. The tables contain the reconstructed average concentrations of PHEs under the IGB conditions. The maximum average baseline concentrations of PHEs were observed in the city of Nizhniy Tagil. In all cases, a significant correlation was established between the concentrations of Fe and PHEs under IGB conditions (significant coefficient A in the model (Eq. 1)). In the figures and tables it can be seen that the cities differ greatly in the average concentration of Fe in contemporary surface sediments. The highest value is observed in Nizhniy Tagil, the lowest – in Nizhniy Novgorod.

**Figure 9:**
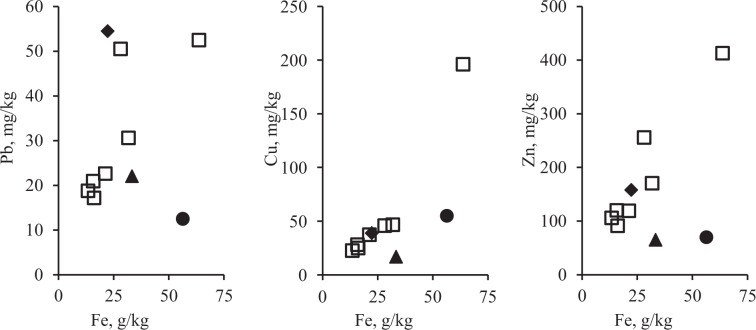
Dependence of average initial baseline PHE concentrations on the average concentration of Fe (squares). Diamonds – abundances in urban soils ^30^, triangles – abundances in floodplain sediment ^31^, circles - Clark according to Taylor ^29^.

The dependence of average concentrations of PHE under IGB conditions on the average concentration of Fe in cities is shown in Figure 9. Figure 9 also shows the Clarke contents of elements according to Taylor^29^, and abundances of PHE and Fe in urban soils and in floodplain sediment according to Alekseenko and Alekseenko^30^ and Salminen^31^ respectively. In this figure, the three points with maximum Fe concentrations belonged to cities of the Ural region (Nizhniy Tagil, Magnitogorsk, and Chelyabinsk), while the three points with minimum Fe concentrations were from cities located on the banks of large rivers (Tyumen, Nizhniy Novgorod, and Rostov-on-Don). The concentrations of Fe and Cu in USDS in most cities were below the Clarke. In contrast, Pb and Zn concentrations were generally higher than the Clarke value.

**Figure 10:**
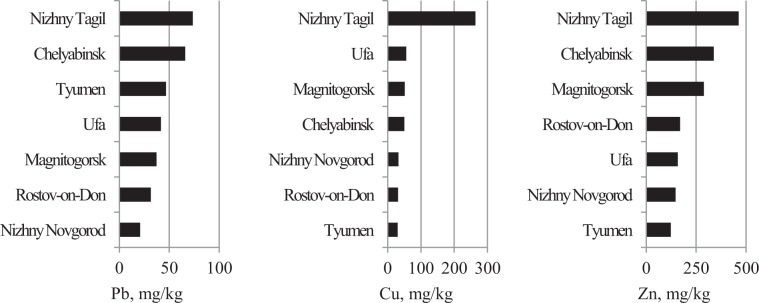
Ranking of cities by the average concentration of PHE.

**Figure 11:**
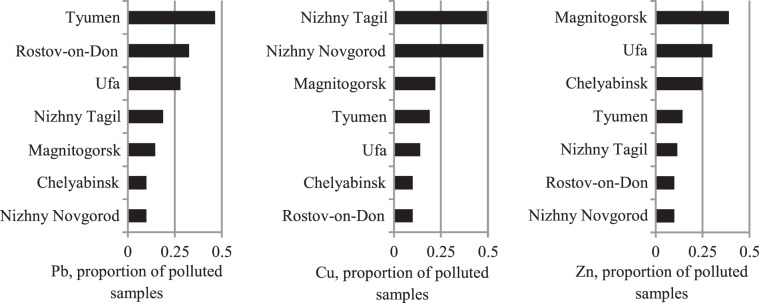
Ranking of cities by the proportion of polluted samples.

**Figure 12:**
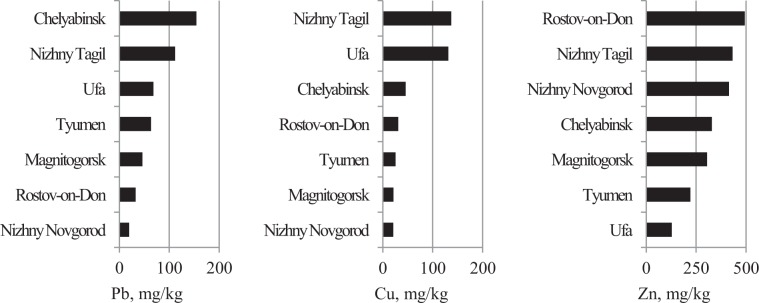
Ranking of cities by the average pollution of polluted samples.

**Figure 13:**
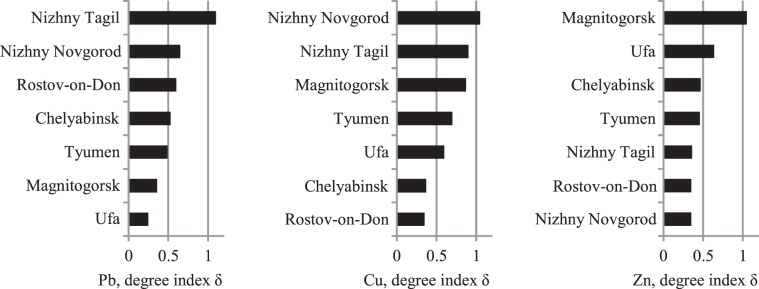
Ranking of the surveyed cities by the degree index δ used in the reconstruction of the IGB level.

**Figure 14:**
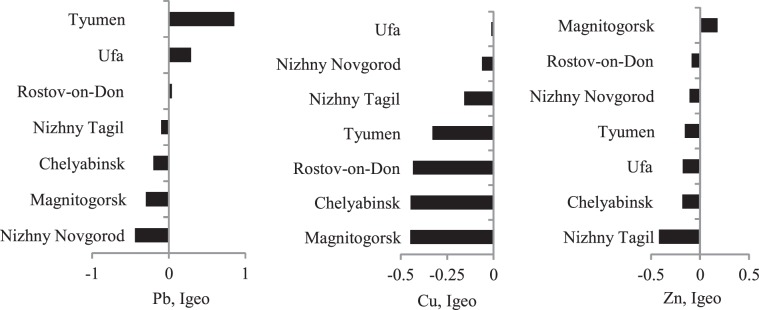
Ranking of the surveyed cities by Igeo.

Figures 10–14 rank the surveyed cities according to various characteristics obtained with regard to their IGB reconstruction. Figure 10 shows the ranking of cities by average sample PHE concentrations. Figures 11 and 12 show the ranking of cities in terms of the percentage of samples classified as polluted and the average pollution of those samples, respectively. The ranking of the cities for these indicators varies considerably. Figure 13 shows the ranking of the surveyed cities in terms of the degree δ-index used in the reconstruction of the IGB level. Figure 14 shows the rankings by the coefficient Igeo taking into account the pollution of the surface sediment with PHE relative to IGB.

## Discussion and Conclusion

The main problem associated with studying the geochemical and environmental roles of surface sediments in urban environments is the lack of criteria for assessing the degree of geochemical transformation and pollution. As a number of authors noted, there are no background objects for urban road deposited sediments that can be used for reference purposes^32^. Absence of the background chemical composition makes it impossible to apply geo-accumulation index Igeo, which is usually used as the indicator of the geochemical transformation. Comparison of PHE concentrations in the urban soils and sediment with the Clarke value is unjustified due to strong influence of anthropogenic processes on formation of these media. In most countries, particularly in Russia, the permissible concentrations and other limits used for environmental management have not been established for USDS.

In the urban environment, some lithologically inherited geochemical contents of major and trace elements in the surface sediment material can be established, which correspond to the baseline concentrations. In this paper, the term initial geochemical baseline (IGB) is used to refer to baseline concentrations. Abundances of chemical elements in USDS and urban soils observed at certain time reflect IGB and pollution occurred in the urban environment to that moment. In particular, concentrations above IGB of typical urban pollutants such as Pb, Cu, and Zn are expected.

A reliable IGB assessment approach is essential for developing environmental geochemistry methods and conducting urban environmental studies. Considering likelihood of appearing both direct substitution of two elements in certain minerals and steady relationship between minerals in soil and sediment formation, the linear regression model can quantitatively describe relationship between concentrations of two elements in different samples^23,33^. The approach with determination of lithologically inherited linear regression between Pb and Al content in natural subsoils was applied in investigations of the topsoils pollution in the Netherlands^34^. As suggested early by Seleznev *et al*.^19^ linear regression model may be also used for describing IGB association between PHE and such conservative lithogenic elements (CLE) as Fe, Al and Mg in USDS.

The patterns of PHEs in relation to CLE in seven cities situated in different climatic and geographical zones and with different patterns of anthropogenic influence are presented in Figures 6–8. According to the pollution model proposed early by Seleznev *et al*.^19^, a cloud of points scattered around the baseline describes the IGB relationship between the concentrations of PHEs and CE. This group of points corresponds to the IGB and any increase in the concentration of PHE in individual samples is supposed to be caused by anthropogenic pollution. In the current study, using mathematical approach suggested early^19^, the parameters of the IGB linear relationship between PHE and Fe concentrations with standard errors (SE) are estimated from sample survey data.

Reconstruction of IGB in form of linear regression allows to estimate both well-known geo-accumulation index and some new indexes which reflect deviation of USDS geochemical composition from IGB in studied cities. Important feature of applied method is possibility to identify polluted samples, in which PHE concentration significantly deviate from IGB regression line. Identification of polluted samples makes it possible to estimate percentage of polluted samples in the sample population and average PHE concentration above IGB in polluted samples. Overall degree of geochemical transformation and deviation from IGB conditions can be described by degree index δ. It appears that the environmental rank of a city significantly varies depending on whether the criterion for ranking is Igeo or suggested new indexes.

In the current study, Fe is selected as the reference CLE. Of course, Al, Mn, or other major elements are useful as reference CLEs in the model. Moreover, some trace elements, such as U, also meet the requirements for use as reference elements. There are many advantages of Fe as a reference CE. Iron is the most abundant metal and is represented in various types of bedrock, so Fe supply to the USDS is possible from multiple sources. Iron has a high mass concentration. Potential technogenic emissions of iron result in concentrations many times higher than would occur from natural sources alone. Chemical analysis methods also exist to accurately and precisely determine Fe concentrations. Aluminium and manganese were tested as reference elements in the city of Ekaterinburg and the consistency of the reconstructed baseline PHE concentration was shown^25^.

Iron concentration differences indicate different IGB levels in the cities examined. The geological environment of the cities is artificial, though it can be assumed that local building materials contain substantial mineral material from local deposits. Technogenic materials, such as slag, are also present in building materials and backfills. The maximum average concentration of Fe is found in Nizhniy Tagil, a city located in the Middle Urals. The impulse to the development of this region was given by the high Fe content in local ore deposits. At present, a large number of enterprises producing enriched iron ore and ferrous metallurgy are concentrated in Nizhniy Tagil and produce slag as a byproduct that is used in road construction and landscaping. Such building materials likely contain elevated Fe concentrations. Also, relatively high Fe concentrations are found in Chelyabinsk and Magnitogorsk where the ferrous metallurgy industry is well developed. In Nizhniy Novgorod, Rostov-on-Don, and Tyumen, the average Fe content of USDS is less than 1.6%. These cities are located in areas with alluvial Quaternary deposits associated with major rivers (Volga, Don, and Tura, respectively).

The calculated values of IGB concentrations of PHE and Fe are mostly different from their Clarke values^29^, abundances in urban soils^30^ and floodplain sediments^31^. In particular, in most cities the concentration of Fe in the surface sediment is below the Clarke value in the Earth’s crust and approximately corresponds to the content in floodplain sediments^31^ and urban soil^30^. Reduced iron content distinguishes the urban environment^3^. The IGB concentrations of Cu and Zn in all cities examined are approximately the same or insignificantly higher than their abundances in floodplain sediments. Lead content is higher than the Clarke value in both floodplain and urban sediments. In contrast, reconstructed initial baseline Pb concentrations are significantly lower than abundances in urban soils. Thus in general, the IGB levels obtained correspond to the abundances of the PHEs in various geological formations.

Determining the objective meaning of the δ-index used for weighting when estimating IGB levels is of broader interest (Tables 3–5, Figure 13). Seleznev *et al*.^19^ assumed that this index relates to a qualitatively defined degree of pollution in the territory under consideration. The degree index δ, in general, correlates with the percentage of polluted samples for all three metals examined (Tables 3–5, Figures 11, 12). A multifactor analysis of the relationship between the δ-index and two quantitative parameters indicates a closer association for Pb and Cu. For Pb, inclusion of the average extent of pollution in polluted samples (Figure 12) as a second factor slightly increases the correlation coefficient for the two-factor model compared to the single-factor model. A similar result is obtained for Cu after excluding Nizhny Tagil from consideration. Some correlation between the concentrations of Fe and Cu in polluted samples is observed in samples from Nizhny Tagil. For Zn, the association of the degree index δ with the average extent of pollution in polluted samples is not found. Thus, the δ coefficient is closely related to the percentage of polluted samples and to some extent with average PHE concentration above IGB in polluted samples. Therefore, the weighting degree index δ can be used as an integral indicator of pollution degree.

Though the geology, climate, and industries in the cities surveyed vary, the mechanisms of USDS formation are similar since the residential areas possess the same basic design and construction features. It is assumed that USDS samples containing particle material from different landscape sites reflect the geochemical conditions of a given block. The use of the same object, namely, surface deposited sediments, and conducting geochemical studies using the same methodology allows for comparison of results from different cities. The sampling of different natural soil types in different cities provides less opportunity for comparative analysis of geochemical conditions anthropogenic transformation.

One of the aims of this study was to analyze methods for ranking cities by the pollution degree. The most direct method of such ranking is presented in Figure 10, which shows the average concentration of three PHEs in USDS samples. A similar approach is routinely used in environmental management practices. For example, the Russian Federation has developed a national project, Ecology, in which the cities of Nizhniy Tagil, Chelyabinsk, and Magnitogorsk are ranked among the cities with the most damaged environment according to total emissions of pollutants into the atmosphere and soil. The average levels of USDS Pb, Cu, and Zn pollution obtained in this study confirm the high ranking of these cities.

PHE concentrations in environmental samples are partially determined by their IGB levels, which differ from city to city. Ranking cities by the percentage of polluted samples results in high rankings for the cities of Tyumen, Ufa, Nizhniy Novgorod, and Rostov-on-Don in which more than 20% of samples are polluted with certain metals. Accounting for the IGB level allows for the estimation of PHE concentration due to pollution. The ranking by this parameter changes the order of most polluted cities. The most contrasting example is for Zn. If the degree index δ is used, the environmental rating of the cities changes even more relative to the use of the observed concentrations alone. The most polluted city indicated by these two methods of ranking differs for two of three metals. Ranking in terms of the degree index δ reflects the difference in the degree of geochemical transformation of the urban environment by the corresponding metal. At the same time, this indicator does not closely correlate with the potential environmental risk associated with these metals.

Another indicator that characterizes the degree of anthropogenic transformation in the urban environment relative to the IGB is the geo-accumulation index Igeo (Figure 10) that provides a ratio of the average observed concentration to the average metal concentration under the IGB level. This coefficient is weakly correlated with degree index δ since Igeo does not take into account the percentage of polluted samples. Igeo does though allow for comparison of metals according to the associated environmental geochemical transformation. For example, in Magnitogorsk, Igeo varies from < 0 for Cu to > 0 for Zn. In Tyumen, Igeo for Pb is > 1, while for other metals it is < 0. Thus, advantage of Igeo is determination of a priority pollutant involved in geochemical transformation for each city.

The suggested ranking methods allow for consideration of the degree of health risks and also changes in geochemical conditions. Such analyses are necessary for predicting future environmental states while taking into account the potential for additional pollutant emissions. Contemporary sedimentation processes provide data for assessing the dynamics of changes in health risks associated with geochemical transformation of the environment. USDSs are one of the objects most sensitive to geochemical transformation. Therefore, geochemical study of USDS allows for more rapid identification of trends in urban environmental states.
